# Higher working memory capacity and distraction-resistance associated with strategy (not action) game playing in younger adults, but puzzle game playing in older adults

**DOI:** 10.1016/j.heliyon.2023.e19098

**Published:** 2023-08-13

**Authors:** Joe Cutting, Bethany Copeland, Fiona McNab

**Affiliations:** a Department of Computer Science, University of York, YO10 5DD, UK; b Department of Psychology, University of York, YO10 5DD, UK

**Keywords:** Gaming, Working memory, Attention, Ageing, Video games

## Abstract

Superior attention and Working Memory (WM) have been reported for habitual action video gamers compared to other gamers or non-players. With an online experiment we measured visuo-spatial WM capacity and ability to ignore distraction, and participants listed the video games they played. Categorising the 209 young adult participants (18–30 years) according to the game type they predominantly played revealed superior WM capacity for strategy and action gamers compared to non-players. However, re-categorising the games according to their constituent game types revealed superior WM capacity and distraction resistance associated with strategy but not action game components. In contrast to younger adults, data from 181 older adults (60–81 years) showed superior WM capacity and distractor-resistance for puzzle gamers, which was equivalent to that of younger adults. The results highlight the need to consider component game types in games research and inform the design of age-appropriate cognitive interventions.

## 1 Introduction

Cross-sectional research has reported that video gamers, and particularly action video gamers, outperform non-players in various aspects of cognition [[1], [2], [3], [4]]. Action video games involve simultaneously juggling various tasks (e.g. detecting, tracking and avoiding certain stimuli), and speeded perceptual processes, which may push the limits of attention, benefiting cognition [3]. A meta-analysis of studies that compared habitual action gamers (predominantly players of first and third person shooter games) with non-action gamers, found the most robust effects on measures of perception, spatial cognition (including spatial Working Memory (WM) tasks) and top-down attention [1] (including ignoring distraction; which requires selective attention). Even when employing broader definitions of action games (for example, including racer games and action adventure games), action gaming has been associated with superior attention and WM [3,5]. Both habitual action gamers, and non-gamers who trained on action games, have shown superior WM performance [[6], [7], [8]]. Similarly, superior attention in action gamers may underlie findings of superior WM in this group [6], or superior learning [9].” Here, we focus on spatial cognition and top-down attention.

A concern for video game research is difficulty categorising games due to an increasing number of hybrid games [10]. If the action games played by participants also contain other game elements (e.g. strategy and puzzle elements), comparisons between action gamers versus other groups may be obscured. Strategy game elements are of particular interest. Training on strategy games has improved cognitive flexibility [11] and WM in older adults [12]. Our analysis took into account that video games often involve elements of multiple game types. A group of volunteers familiar with video games identified the game types present in each game listed by the participants, and participants were categorised according to the combination of component game elements in the games they played. We then compared Working Memory Capacity (WMC) and distractor-resistance between these groups, to address whether the reported superiority of action games holds with more fine-grained game categorisation.

Boot et al. [13] also addressed whether attention benefits are specific to action games compared to strategy or puzzle games, although not with the approach of categorising games according to their component game elements. Building on the approach of Green & Bavelier [3], they directly compared the effects of playing 21 h of action, strategy or puzzle video games on various measures of attention. However, failing to replicate Green & Bavelier [3], they saw no benefits for any of the groups, and no significant difference between habitual action gamers and non-gamers in three attention tasks (an attentional blink task, an enumeration task and a functional field of view task; although note that other tasks, including a visual short term memory task did show a significant difference). Although differences in task parameters might account for this, another possibility is that the inclusion criteria for the “non-gamer” control group, who played for 1 h a week or less, is responsible. If at least part of the action gamer advantage in attention can be attributed to pre-existing differences between those who choose to play action video games, compared to those who choose not to, the inclusion of participants who play up to an hour per week in the “non-gamer” group may have diluted group differences. We therefore restricted our “non-gamer” group to those who reported playing no video games.

Although some studies have taken an intervention approach to try to isolate the effects of video gaming from other differences between gamers and non-gamers ^(e.g. 3, see also 13)^, our approach was to focus on more fine-grained analysis of habitual versus non-game players. By building a more detailed understanding of how WM and attention differ between players of specific combinations of game elements, and how these associations may be affected by age, we aim to facilitate more targeted intervention studies.

Although many studies have addressed how video gaming relates to different aspects of attention ^(e.g. 3)^, we used measures of attention that are particularly relevant for WM. The effective ignoring of irrelevant information predicts good WM performance and may form a basis for WM capacity [14,15]. We measured participant's ability to ignore two types of distraction; distraction that is presented together with the items that are to be remembered (Encoding Distraction, ED) and distraction that is presented during WM maintenance (ie. during the WM delay period, Delay Distraction, DD). Ability to ignore these different types of distraction separately predicts WMC, suggesting that they involve separate mechanisms [16,17]. With the same visual spatial WM task we measured WMC, as well as the participant's ability to ignore ED and DD.

Age-related changes in WM from late adolescence/early adulthood are well established [[17], [18], [19], [20]]. Furthermore, the ability to ignore ED and DD seem to be separately affected by ageing, and the way they shape WMC also seems to change with increasing age [17]. If playing different types of video game is associated with superior performance in different aspects of attention, it is possible that the type of video game associated with superior WM performance might differ between younger adults and older adults. Wang el al [5]. reported a meta-analysis of intervention studies which showed a larger impact of video gaming in young compared to older adults, but it is not clear whether cognitive differences between players of different game types are consistent between younger and older adults. Kim et al. [21] on the other hand found superior performance for early middle-aged gamers compared to age-matched non-gamers on the Tower of London Task (TOL), but no significant difference was shown by younger adults. Age effects have been difficult to research as recruiting older adults who are habitual video gamers has been problematic. Bediou et al. [1] found no cross-sectional studies of older adults (above 65 years) which met the inclusion criteria for their meta-analysis. By using an online experiment, we were able recruit enough older adults to examine WM and distractor-resistance in habitual strategy or puzzle gamers and non-gamers, and directly compare younger and older adults.

We therefore investigated whether WMC and resistance to ED and DD, differed between puzzle, strategy or action gamers and non-gamers. We addressed whether the superiority of action games reported in the literature remains when games are categorised by their constituent game elements. We also compared groups of younger and older adults to address whether the game-types associated with superior WMC and distractor-resistance vary with age.

## 2 Methods

### 2.1 Participants

543 participants completed the experiment, which was approved by the Psychology Department Ethics Committee at the University of York (approval number 906). All participants gave informed consent. The data from 457 participants were collected using Prolific (www.prolific.co; accessed June 2021). The data from 86 participants were collected as part of an undergraduate practical session on statistics. Students shared the link for the online experiment. Data from 14 participants were excluded from the analysis as they reported that they were colour blind. Data from a further 12 participants were excluded because they reported technical difficulties (for example, their device restarted). Data from one participant was excluded because they listed games but reported playing for zero hours a week, data from 2 participants were excluded as they did not specify the number of hours they played video games and data from 31 participants were excluded because they reported playing video games, but did not provide the names of any video games. Data from one further participant was excluded because they failed at the easiest level of one of the conditions (ie. failed two consecutive trials of WM load 2). Data from 482 participants remained for analysis (ages 18–81; 297 female, 177 male, 6 non-binary and 2 other; 409 recruited through Prolific and 73 recruited through undergraduate students sharing the link).

### 2.2 Experimental design and task

As part of the online experiment participants were asked how many hours a week they spend playing digital games (including games on their phone, computer or gaming console), and the year in which they started playing digital games such as arcade games, PC games, mobile games etc. (if applicable). They were also asked to list the digital games they played in the last week (to avoid errors associated with participants trying to remember the games they had played over a longer period). The questions are listed in the Supplementary Material.

**Fig. 1:**
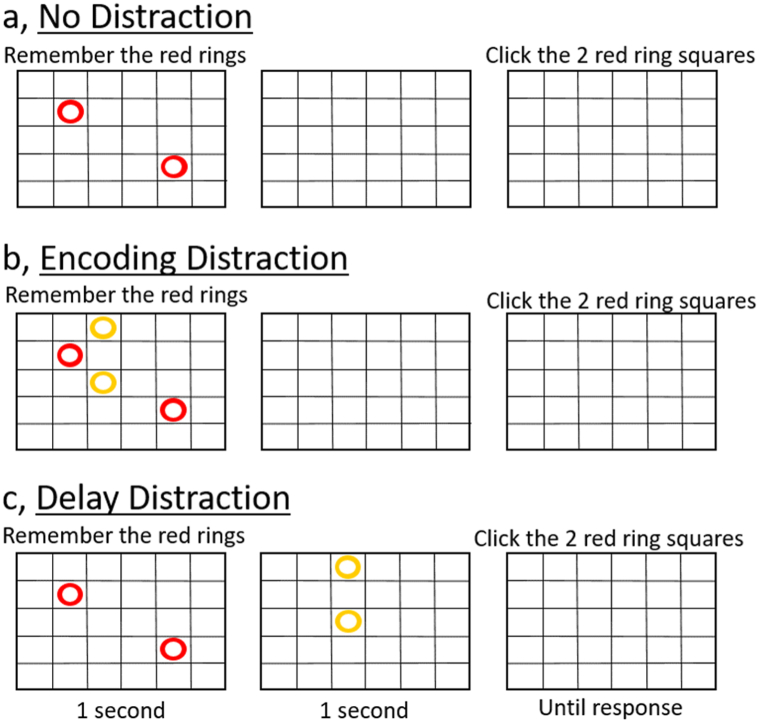
The working memory task. Participants were asked to remember the positions of the red circles and ignore any yellow circle distractors. In the No Distraction condition (a) there were no yellow circles. In the Encoding Distraction condition (b), two yellow circle distractors were presented together with the red circles and in the Delay Distraction condition (c), two yellow circle distractors were presented after the red circles had disappeared.

They then completed the online WM task (Fig. 1). There were seven conditions, three of which are relevant to this study. Participants were asked to remember the positions of red circles that appeared on a 5 × 6 grid for 1s. At the end of each trial they were presented with an empty grid and asked to press on the grid positions in which red circles had appeared. They were informed immediately when they made a mistake. For all conditions there was a delay period of 1s during which time the grid was shown, after the red circles had disappeared and before the participants could make their response. In the “No Distraction” condition (Fig. 1a) only red circles were displayed. In the “Encoding Distraction” condition (Fig. 1b) two yellow distractor circles were displayed together with the red circles. In the “Delay Distraction” condition (Fig. 1c) two yellow distractor circles were shown during the delay period. For each condition the number of red circles (WM load) started at two, and increased with performance (one red circle was added each time a trial was answered correctly) until the participant failed two successive trials of a condition (from which point the game continued without that condition). When there were no conditions left, the WM task immediately started again, with the WM load reverting to two circles to remember for each condition. In this way participants worked through the WM task twice, and the first time was treated as a practice.

**Fig. 2:**
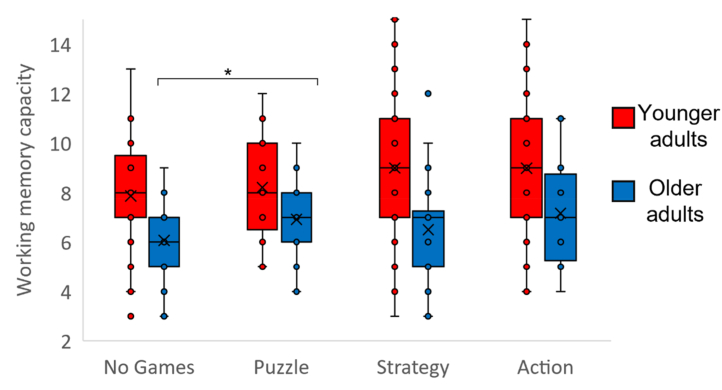
Box-and-whisker plot of working memory capacity for each game group for younger (red) and older (blue) participants. First and third quartiles are delineated by boxes. Whiskers represent the maximum and minimum values within 1.5 times the interquartile range from the first or 3rd quartile. Crosses show the mean for each group. Groups were defined by the game type that featured most in the games listed by the participants. As we had not observed a significant interaction between game group and age, we applied a Bonferroni correction for the nine comparisons reported, such that p < 0.006 was considered statistically significant (indicated by *).

### 2.3 Data analysis

We only considered data from the second time each participant completed the WM task. Performance in each condition was measured as the last WM load at which a trial was answered correctly. Performance from the No Distraction condition was used as a measure of WMC. There was no indication that estimates of WMC differed between participants who were recruited by the different methods, when controlling for age (main effect of recruitment method: F (1,479) = 0.039, p = 0.843), so data were pooled across these two methods of recruitment.

To confirm that individual differences in the ability to ignore ED and the ability to ignore DD uniquely predicted individual differences in WMC, as we have previously reported [16], we used the regression model ND = α + β_1_ ED + β_2_ DD, where ND, ED, and DD represent WM scores for the ND, ED, and DD conditions respectively, α is the intercept and β_1_ and β_2_ are the regression coefficients. Performance in the ED and DD distractor conditions were used to predict performance in the No Distraction condition (ie. WMC). We examined the unique contributions of ED and DD.

#### 2.3.1 Measures of ED and DD resistance ability

We also used regression analysis to estimate ED and DD resistance ability for each participant [16,22]. For ED resistance ability, we used the residual in WM performance in the ED condition after accounting for WM performance in the DD condition, using the regression model ED = α + β DD, where WM scores for the ED condition were taken as the dependent variable while WM scores for the DD condition were the predictor. Similarly, for DD resistance ability we used the residual in WM performance in the DD condition after accounting for WM performance in the ED condition using the regression model DD = α + β ED, where WM scores for the DD condition were the dependent variable while WM scores for the ED condition were used as a predictor. In these regression models, the residuals for both ED and DD conditions were estimated for each individual by calculating the differences between the observed WM score and the predicted WM score for each condition since the residual indicates the variance that is left over in the data; that is, the variability that could not be explained by the other type of WM distraction. In this way, our measures of ED and DD resistance ability represent the unique variance associated with ignoring that type of distraction, controlling for shared variance associated with WM for relevant stimuli and instructions to ignore yellow circle distractors.

#### 2.3.2 Participant categorisation

A group of volunteers (all undergraduate students studying Psychology at the University of York) worked together to code the games that were listed by the participants, without sight of the WM performance data. A puzzle game was defined as focussing “on logical and conceptual challenges … [focussing] on puzzle solving as its primary gameplay activity” [23]. A strategy game was defined as involving “planning and coordination of a set of operations in order to effectively reach a goal” [24]. An action game was defined as a “game characterized by simple action and response gameplay … the defining characteristic is that enemies and obstacles are overcome by “physical” means rather than involves intellectual problem solving” [25]. Note that this definition of action games, which is broader than that used by Bediou et al. [1], but consistent with those used by others [3,4,5], was chosen to enable us to compare action games with and without strategy game elements. The games listed by participants, and the way they were coded, are shown in the Supplementary Material. When there was an action element in a game, the volunteers gave each game an “action rating” according to whether over half of the time spent playing was likely to be “down time” (time with no/minimal action), approximately half of the time was likely to be “down time” or less than half of the time was likely to be “down time”.

Firstly game-playing participants were categorised as being predominantly Action, Strategy or Puzzle game players (according to the number of games they listed that featured each game-time, as well as the extent to which each game type featured in the games that were listed). We then categorised the participants according to which elements were present in the games they listed, for example *Action & Strategy*. The group of volunteers discussed each participant/game and reached a consensus for each classification. This was done in four sessions, with at least five volunteers contributing to each session.

#### 2.3.3 ANOVA and t-tests

For each type of categorisation, an ANOVA was run to compare WMC and distractor resistance between game groups. To examine any differences between age groups, between-subjects ANOVAs were also run which included the factors game group and age group (Younger: 18–30 years; Older: 60+ years). When significant effects were observed, these were followed up with independent samples t-tests, without a Bonferroni correction. When there was no significant game group * age group interaction revealed by the ANOVA, we applied a Bonferroni correction to any further t-tests.

All statistical analyses were performed using IBM SPSS Statistics 20, and two-tailed tests. *P* values less than 0.05 were considered to be significant. Data will be shared using the APA data repository. The list of video games reported by participants, and the way they were coded, are listed in the Supplementary Material.

#### 2.3.4 Power analysis

Following their meta-analysis of cross-sectional studies, Bediou et al. (2020) reported Hedges effect size estimates of 0.75 and 0.625 for spatial cognition and top-down attention respectively. We estimated (G*Power; http://www.gpower.hhu.de/en.html) that, with an effect size of 0.65, a sample size of 20 would be needed to achieve statistical power of 80%, assuming a ratio of 3:1 participants in the non-gamer/gamer groups respectively. Therefore, groups with fewer than 20 participants were excluded from further analysis.

## 3 Results

### 3.1 Predominant game type

Assigning participants to groups based on the game type that featured most in their list of games played resulted in the groups described in Table 1. As there were fewer than 20 older participants in the Action group, data from this group were omitted from the analyses as it was likely that these comparisons would be underpowered [1].

**Table 1:** The number of participants in each group when the game-playing groups were defined by the game type that featured most in the games listed by the participants. The No Games group included participants who reported playing for zero hours a week and listed no games. The younger participants were 18–30 years and the older participants were 60–81 years old.

	No Games	Puzzle	Strategy	Action
Younger participants	53	33	43	80
Older participants	88	59	34	12

**Table 2:** The results, from younger and older participants, of independent samples t-tests comparing WMC between groups when game-playing groups were defined by the game type that featured most in the games listed by the participants. Degrees of freedom are shown in brackets. † indicates that equal variances were not assumed, as indicated by the results of Levene's test. As we had not observed a significant interaction between game group and age, we applied a Bonferroni correction for the nine comparisons reported, such that p < 0.006 was considered statistically significant.

	Puzzle			Strategy			Action		
	t	p	Cohen's d	t	p	Cohen's d	t	p	Cohen's d
Younger adults									
No Games	−0.75 (84)	0.453	−0.17	−2.28 (94)	0.025	−0.47	−2,73 (126.278)†	0.007	−0.46
Puzzle				−1.38 (74)	0.171	−0.32	−1.53 (111)	0.130	−0.32
Strategy							0.03 (121)	0.980	0.01
Older adults									
No Games	**−3.62 (145)**	**<0.001**	**−0.61**	−1.20 (45.826)†	0.236	−0.28			
Puzzle				1,17 (91)	0.245	0.25			

Considering only the younger participants, there was a significant main effect of game group (F(3,205) = 2.93, p = 0.035, η^2^ = 0.04). As shown in Fig. 2 and Table 2, for younger adults there was significantly greater WMC for the Strategy and Action groups, but not the Puzzle group, compared to the No Games group. There was no significant difference between any of the game-playing groups.

### 3.2 Comparison between age groups

To directly compare the two age groups, we considered all groups except the Action game group. As expected there was significantly lower WMC for the older age group than the younger age group (F (1,304) = 67.73, p < 0.001, η^2^ = 0.18). There was also a main effect of game group (F (2,304) = 4.97, p = 0.008, η^2^ = 0.03), but no significant interaction between age group and game group (F (2,304) = 2.05, p = 0.131, η^2^ = 0.01). Adding the number of hours spent playing games as a co-variate did not affect any of these results (main effect of age group: F (1,303) = 66.57, p < 0.001, η^2^ = 0.18; main effect of game group: F (2,303) = 5.42, p = 0.005, η^2^ = 0.04; interaction between age group and game group: F (2,303) = 2.34, p = 0.098, η^2^ = 0.02, effect of number of hours: F (1,303) = 1.30, p = 0.255, η^2^ < 0.01; see below and Fig. 5 for the mean number of hours played). Although this interaction did not reach statistical significance, the pattern of results was different for the two age groups, as shown in Fig. 2 and Table 3. Whereas the younger participants had shown significantly greater WMC for the Strategy group compared to the No Games group, for older participants there was significantly greater WMC for the Puzzle group, but not the Strategy group, compared to the No Games group. As we had not observed a significant interaction, we applied a Bonferroni correction for the nine comparisons reported in Table 2, such that p < 0.006 was considered statistically significant. Whereas the younger participants showed a trend for greater WMC for the Action group compared to the No Games group (p < 0.007), for older participants there was significantly greater WMC for the Puzzle group, but not the Action or Strategy groups, compared to the No Games group.

**Table 3:** The number of participants in each group when the game-playing groups were defined by the component game types that featured in the games listed by the participants.

	No Games	Puzzle	Strategy	Action	Puzzle & Strategy	Puzzle & Action	Strategy & Action	Puzzle, Strategy & Action
Younger participants	53	21	26	20	15	2	76	24
Older participants	88	39	40	1	16	1	10	1

### 3.3 Component game types

Assigning participants to groups based on the game types that featured in their list of games resulted in participant numbers shown in Table 3. As there were fewer than 20 younger participants in the Puzzle & Strategy group and the Puzzle & Action group and fewer than 20 older participants in the Action, Puzzle & Strategy, Puzzle & Action, Strategy & Action and Puzzle & Strategy & Action groups, these groups were excluded from further analysis as, based on the results of Bediou et al. [1], it was likely that analyses involving these groups would be statistically underpowered.

**Fig. 3:**
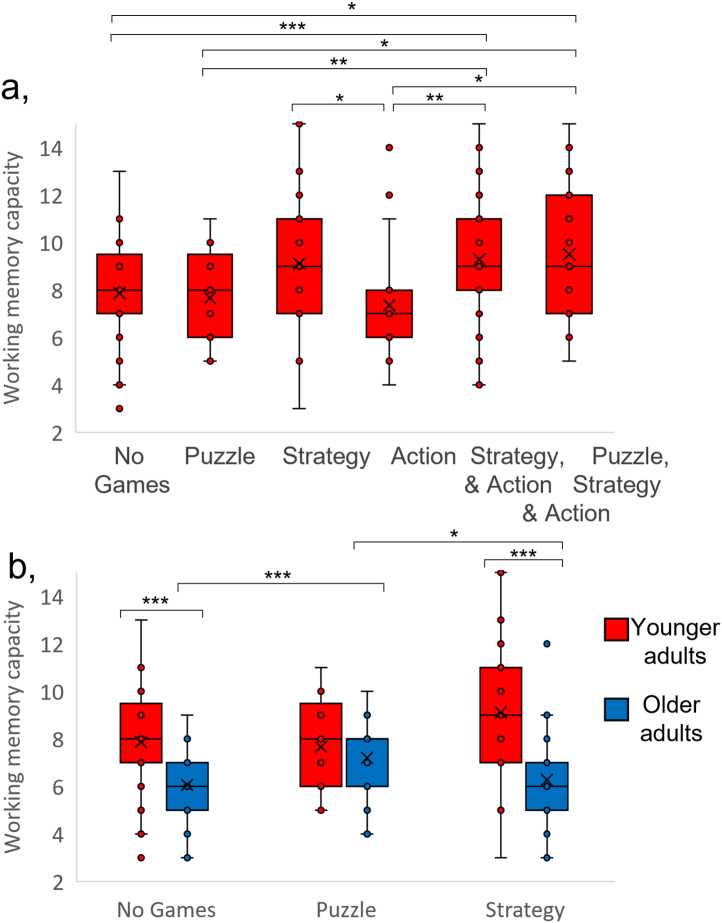
Box-and-whisker plots of working memory capacity for each game group for younger (a & b; red) and older (b; blue) participants. First and third quartiles are delineated by boxes. Whiskers represent the maximum and minimum values within 1.5 times the interquartile range from the first or 3rd quartile. Crosses show the mean for each group. Groups were defined by the component game types that featured in the games listed by the participants. * indicates p < 0.05, ** indicates p < 0.01, *** indicates p < 0.001.

We first compared WMC between each of the remaining groups for younger adults. There was a significant main effect of game group (F (5,214) = 4.78, p < 0.001, η^2^ = 0.10; Fig. 3a). Adding number of hours a week spent playing games as a co-variate did not affect this result (main effect of game group: F (5,213) = 3.71, p = 0.003, η^2^ = 0.08; effect of number of hours: F (1,213) = 0.04, p = 0.852, η^2^ < 0.01). The results of independent samples t-tests between the groups are shown in Table 4. There was no significant difference between WMC for the No Games group and any of the single-game groups, although the greater WMC for the Strategy group compared to the No Games group would have reached significance with a one-tailed test. Similarly, WMC was greater for the Strategy group than the Puzzle group but this just failed to reach statistical significance with a two-tailed test. However, there was significantly greater WMC for the Strategy & Action group and the Puzzle, Strategy & Action group compared to the No Games group, the Puzzle group and the Action group, but not the Strategy group. The inclusion of a strategy component was associated with superior WMC for action game players, but the inclusion of an action component was not associated with superior WMC for strategy game players. Similarly, the inclusion of strategy and action game components was associated with superior WMC for puzzle game players. These results are consistent with superior WMC associated specifically with playing games that include a strategy game component.

**Table 4:** The results of independent samples t-tests comparing WMC between groups, with groups defined by the component game types in the games that participants reported playing. Degrees of freedom are shown in brackets, and † indicates that equal variances were not assumed, as indicated by the results of Levene's test.

	Puzzle			Strategy			Action			Strategy & Action			Puzzle, Strategy & Action		
Younger Adults	t	p	Cohen's d	t	p	Cohen's d	t	p	Cohen's d	t	p	Cohen's d	t	p	Cohen's d
No Games	0.39 (72)	0.701	0.10	−1.91 (37.487)†	0.063	−0.52	0.89 (71)	0.375	.23	**−3.50 (127)**	**<0.001**	**−0.63**	**−2.49 (34.39)**†	**0.018**	**−0.69**
Puzzle				−1.95 (45)	0.058	−0.57	0.47 (39)	0.644	0.15	**−2.89 (95)**	**0.005**	**−0.71**	**−2.58 (39.356)†**	**0.014**	**−0.75**
Strategy							**2.13 (44)**	**0.039**	**0.63**	−0.30 (100)	0.764	−0.07	−0.46 (48)	0.646	−0.13
Action										**−3.20 (94)**	**0.002**	**−0.81**	**−2.61 (42)**	**0.012**	**−0.79**
Strategy & Action													−0.36 (98)	0.721	−0.08
Older Adults															
No Games	**−4.32 (125)**	**<0.001**	**−0.83**	−0.73 (126)	0.466	−0.14									
Puzzle				**2.56 (77)**	**0.012**	**0.58**									

### 3.4 Comparison between age groups

For the No Games, Puzzle and Strategy groups there were more than 20 participants in younger and older age groups, so we were able to directly compare age groups (Fig. 3b). As expected we observed a significant main effect of age group, with lower WMC associated with older participants (F (1,261) = 46.59, p < 0.001, η^2^ < 0.15), and a significant main effect of game group (F (2,261) = 3.72, p = 0.026, η^2^ < 0.03). However, there was also a significant interaction between game group and age-group (F (2,261) = 6.19, p = 0.002, η^2^ < 0.05). Although adding number of hours a week spent playing games as a covariate gave a significant effect of this variable (F (1,260) = 5.73, p = 0.017, η^2^ < 0.02), it did not affect any of the other results (main effect of age group: F (1,260) = 37.15, p < 0.001, η^2^ < 0.13; main effect of game group: F (2,260) = 6.50, p = 0.002, η^2^ < 0.05; interaction between age group and game group: F (2,260) = 6.34, p = 0.002, η^2^ < 0.05). Whereas younger participants showed superior WMC for the Strategy group (which just failed to reach significance with a two-tailed test), for older participants WMC was significantly greater for the Puzzle group compared to both the No Games group and the Strategy group (Table 4; Fig. 3b).

The superior WMC for older adults in the Puzzle group meant that equivalent WMC was seen for younger and older adults in this game group (t (58) = 1.08, p = 0.285, η^2^ < 0.29), whereas for both the No Games and Strategy groups there was significantly greater WMC for younger compared to older groups (No Games group: t (77.869) = 5.61, p < 0.001, η^2^ < 1.08, equal variance not assumed as indicated by the results of Levene's test (F = 12.63, p < 0.001); Strategy group: t (36.497) = 4.38, p < 0.001, η^2^ < 1.22, equal variance were not assumed as indicated by the results of Levene's test (F = 8.09, p = 0.006).

### 3.5 Distraction

As observed previously [16], individual differences in ED and DD distractor-resistance separately predicted WMC, consistent with separate ED and DD resistance mechanisms which constrain WMC. The model accounted for a significant amount of variance (adjusted r^2^ = 0.42, p < 0.001) and as predicted ED and DD score made a significant contribution in the model (β_1_ = 0.41, β_2_ = 0.33, where β refers to standardized beta; p < 0.001 for each).

Based upon the results for WMC described above, and to maximize the number of participants we could include in each group, we used ANOVAs to compare estimates of ED and DD resistance ability between those who played games with a puzzle, strategy or action component, and those who did not (i.e. they played no games or games without that component). We performed separate ANOVAs for each age group given that estimates of distractor-resistance ability were calculated with separate regression analyses for younger and older participants.

**Fig. 4:**
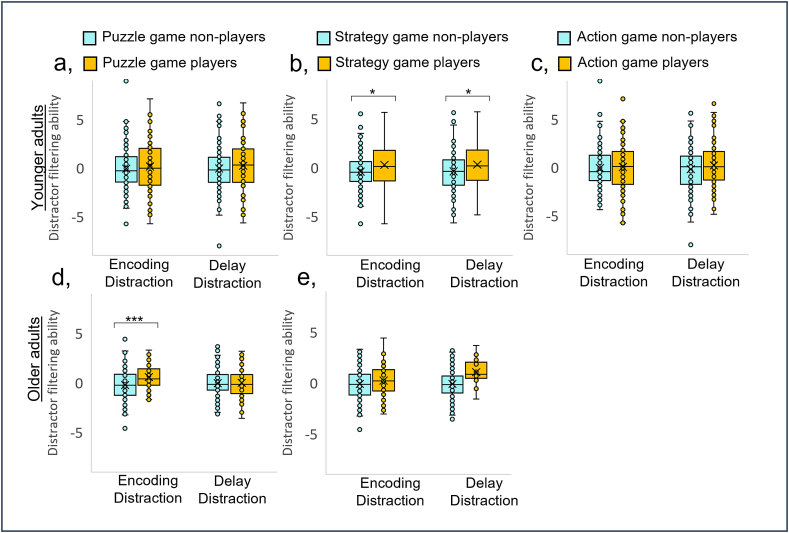
Box-and-whisker plots of estimates of encoding and delay distractor-resistance ability for those who played games with a puzzle (a,d), strategy (b,e) or action (c) component, and those who did not (ie. they played no games or games without that component) for younger (a–c) and older (d–e) participants. First and third quartiles are delineated by boxes. Whiskers represent the maximum and minimum values within 1.5 times the interquartile range from the first or 3rd quartile. Crosses show the mean for each group. * indicates p < 0.05, *** indicates p < 0.001.

As shown in Table 5 and Fig. 4, for younger adults there was a significant main effect of strategy game type (Fig. 4b), but no significant effects of puzzle (Fig. 4a), or action game types (Fig. 4c). Those who played games with a strategy component had greater distractor-resistance ability compared to those who did not. For younger participants this was significant for both types of distractor-resistance (younger/ED: t (229.710) = -2.47, p = 0.014, η^2^ = −0.31 (equal variances were not assumed as indicated by the results of Levene's test: F = 6.61, p = 0.011; younger/DD: t (234) = -2.29, p = 0.023, η^2^ = −0.30; Fig. 4b). For older adults (Fig. 4d–e), although the main effect of game type just reached statistical significance, independent samples t-tests did not reach significance for ED (t (194) = -1.49, p = 0.137, η^2^ = −0.23) or DD (t (194) = -0.82, p = 0.415, η^2^ = −0.12) (Fig. 4e). However, for older adults there was a significant difference between participants who played games with a puzzle component and those who did not, and a significant interaction between game group and distraction type. Older adults who played games with a puzzle component had greater ED resistance ability (t (194) = -3.58, p < 0.001, η^2^ = −0.56), but there was no significant difference in DD resistance ability (t (194) = -0.20, p = 0.842, η^2^ = −0.03) (Fig. 4d).

**Table 5:** The results of ANOVAs comparing encoding and delay distraction resistance ability between participants who reported playing games with a puzzle, strategy or action component and those who did not. For younger participants the degrees of freedom = 1,234, for older participants degrees of freedom = 1, 194).

	Main effect of game type			Main effect of distraction type			Interaction between game type and distraction type		
**Younger Adults**	F	p	*η*^2^	F	p	*η*^2^	F	p	*η*^2^
Puzzle	2.11	0.148	0.01	0.03	0.876	<0.01	0.02	0.903	<0.01
Strategy	**20.27**	**<0.001**	**0.08**	0.01	0.912	<0.01	<0.01	0.998	<0.01
Action	2.71	0.101	0.01	0.01	0.922	<0.01	0.31	0.578	<0.01
**Older Adults**									
Puzzle	**13.66**	**<0.001**	**0.07**	1.01	0.316	<0.01	**4.12**	**0.044**	**0.02**
Strategy	**4.92**	**0.028**	**0.03**	0.09	0.762	<0.01	0.20	0.656	<0.01

**Fig. 5:**
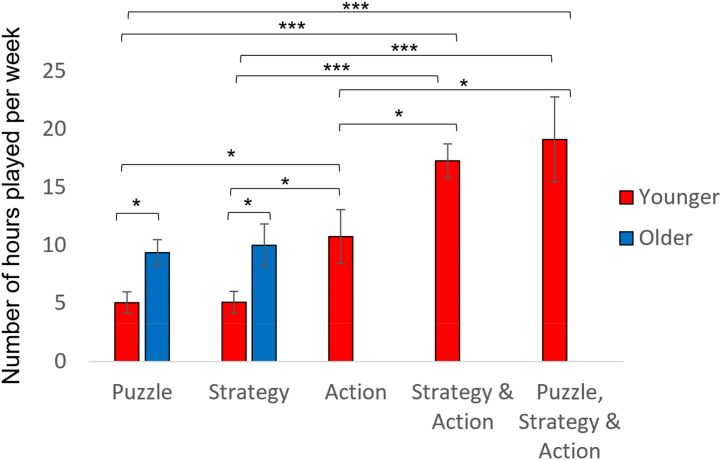
The mean number of hours spent playing video games for each game group, and age group, when game-playing groups were defined by the component game types that featured in the games listed by the participants. Error bars show ±1 standard error. * indicates p < 0.05, *** indicates p < 0.001.

### 3.6 Number of hours played per week

As described above, the between group differences we observed could not be accounted for by differences in the number of hours a week spent playing the different types of video games. Fig. 5 illustrates between group differences in the number of hours played. For younger adults there was a significant group difference in the number of hours played (F (4,162) = 9.57, p < 0.001, η^2^ = 0.19). When considering only the puzzle and strategy single-component groups, we observed that older participants spend more hours playing puzzle and strategy games (F (1,122) = 9.42, p = 0.003, η^2^ = 0.07), and there was no main effect of game group (F (1,122) = 0.05, p = 0.832, η^2^ < 0.01) and no interaction between age group and game group (F (1,122) = 0.04, p = 0.844, η^2^ < 0.01).

### 3.7 Action rating

For younger adults who reported games that had an action component, there was no significant correlation between mean or maximum action rating and WMC, ED or DD resistance ability (p > 0.154).

## 4 Discussion

In line with previous research [1,12], when we categorised participants according to the predominant game type they played, there was significantly greater WMC for younger adult action and strategy gamers compared to non-gamers. The most striking finding from our more fine-grained analysis, which considered the component game types played by participants, was that there was no benefit associated with playing pure action games. For younger participants, equivalent WMC scores were seen for pure action gamers as for non-gamers, or pure puzzle gamers. This result could not be explained by differences in the number of hours participants reported to spend playing video games, as our analysis controlled for this, and participants who played purely action games spent significantly *more* hours playing than those who played purely strategy games. Instead, a significant WMC benefit was seen for players of games with a strategy component. Although the comparison between non-gamers and pure strategy gamers did not reach statistical significance (p < 0.063) with a two-tailed test, those who played purely strategy games had significantly greater WMC compared to those who played purely action games. Furthermore, those who played games with both action and strategy elements had greater WMC compared to those who played purely action games, but not compared to those who played purely strategy games, further indicating that strategy and not action game elements are associated with greater WMC for younger adults. It is possible that the comparison between the non-gamers and purely strategy gamers failed to reach statistical significance due to variability in the non-gamer group, which may have included participants who previously played strategy games, trained their WM in other ways, or would play strategy games given the opportunity.

Perhaps due to our method of recruitment, for most groups there were more female than male participants. However, we did not expect working memory differences between females and males [26] and for groups in which there were comparable numbers of males and females, we were able to confirm this (younger action gamers: t (76) = -1.04, p = 0.300, η^2^ = −0.24; older non-gamers: t (85) = -0.66, p = 0.509, η^2^ = −0.14).

One limitation of our study was that reporting video gaming prior to completing the WM task may have advantaged the gamers, if they expected to do well [27]. We consider it unlikely that this could explain our results for two reasons. Firstly, expectation would have presumably benefited all gamers equally, or benefited action game players to a greater extent, and for younger adults the WMC benefit was specific to players of strategy game elements. Secondly, it seems unlikely that expectation effects could have affected our difference measures (i.e. our estimates of ED and DD resistance), which also showed no benefit linked to action game elements.

Our measures of distractor-resistance; the ability to ignore distraction during WM encoding or maintenance, are particularly relevant to WMC. For both types of distractor-resistance, specifically players of games involving a strategy component showed superior performance, providing a potential mechanism for the superior WMC shown by these players^(see 5)^. Playing action games has been associated with superior performance in various measures of attention, perception and executive function [1,3]. For example, Green and Bavelier [3] found superior performance in measures of attentional capacity, the spatial distribution of attention and the ability to process items over time. Our results indicate that playing action game elements does not predict superior performance for distractor-resistance within a WM task. Instead, this type of attention may be more closely aligned to the process of ignoring distraction while maintaining multiple pieces of relevant information during strategy game play, rather than the high-speed perceptual processing required for action game elements.

If playing video games provides cognitive training, or if those with higher cognitive performance spend longer playing video games, we might have expected a correlation between cognitive performance and the number of hours spent playing video games. However, in line with Unsworth et al. [4], we saw no such association. However, we only asked participants to report on their video gaming during the previous week, and not how long they spent playing each game type. Furthermore, the use of self-report may have introduced errors [[28], [29], [30]], and spending more time on a task does not always improve performance [31]. Future work is needed to interrogate this possible association.

For younger adults we observed superior WMC for strategy gamers compared to non-gamers, but for older adults superior WMC was seen for puzzle gamers. Older puzzle gamers showed equivalent WMC to younger puzzle gamers or non-gamers. Differences in time spent playing did not account for this. Furthermore, older gamers spent significantly longer playing than younger gamers, irrespective of game-type played, but superior WM was seen for puzzle not strategy gamers. Age differences in the level of difficulty of play [32], game culture, acceptance and enjoyment of different game genres may have contributed to the age-differences [33]. Although older adults with superior WMC may be more likely to play puzzle games, it is also possible that playing puzzle games improves WM, and equivalent training gains may be seen for younger and older adults with age-appropriate training (see Wang et al. [5] for results of a meta-analysis showing greater training gains for younger adults playing action video games than older adults).

It is also of interest that older puzzle gamers showed greater distractor-resistance than non-gamers, and this was specific to Encoding Distraction (ED). Resistance to Delay Distraction (DD) has been shown to decline more rapidly with increasing age compared to ED [17]. Therefore, older adults with superior WMC may favour puzzle games if those games place more demands on resisting ED, compared to DD. Alternatively, older puzzle gamers may be more susceptible to training gains in ED compared to DD, and perhaps use strategies which favour mechanisms that support ED resistance.

Although further work is needed to unpick the specific aspects of video games that relate to specific aspects of attention, our results indicate that action video gamers do not show superior performance for all types of WM and distractor-resistance. For younger adults we observed an association between strategy game components and superior WMC and distractor-resistance, irrespective of whether action game elements are included. In contrast, for older adults, superior WMC and distractor-resistance were associated with puzzle gaming. This provides insights for future targeted, age-appropriate interventions.

## Author contribution statement

Joe Cutting: Conceived and designed the experiments; Performed the experiments; Contributed reagents, materials, analysis tools or data; Wrote the paper.

Bethany Copeland: Conceived and designed the experiments.

Fiona McNab: Conceived and designed the experiments; Performed the experiments; Analyzed and interpreted the data; Wrote the paper.

## Data availability statement

Data will be made available in a repository on acceptance.

## Declaration of competing interest

The authors declare that they have no known competing financial interests or personal relationships that could have appeared to influence the work reported in this paper.
