# Supplementary material for: Higher working memory capacity and distraction-resistance associated with strategy (not action) game playing in younger adults, but puzzle game playing in older adults
Source: Heliyon. 2023 Aug 13;9(8):e19098. doi: 10.1016/j.heliyon.2023.e19098 (PMC10448072; doi:10.1016/j.heliyon.2023.e19098)
Supplement: Multimedia component 1 [file mmc1.docx]

Supplementary Material

After completing the working memory task, participants were asked the following questions.

Gender

O Male O Female O Other (Please state)

Age

Do you have any form of colour-blindness?

O No O Yes (please give details)

How many hours a week do you spend playing digital games? (includes

games on your phone, computer or gaming console)

Which digital games have you played in the last week? (write N/A if none)

Roughly which year did you start playing digital games, such as arcade

games, PC games, mobile games etc.? (Write N/A if never played)

The video games reported by participants and the way they were coded.

| 1010 – Puzzle |
| --- |
| 2048 - Puzzle |
| 8 ball pool – Strategy |
| Adventure/HOGs – Undefined |
| AFK Arena New – Strategy & Action |
| Age of Empires 4 – Strategy |
| Airplane Chef – Strategy |
| Angry Birds – Puzzle & Strategy |
| Animal Crossing – Strategy |
| Animal Crossing: New Horizons – Strategy |
| Apex Legends – Strategy & Action |
| Arena of Valor – Action |
| ARK – Strategy & Action |
| Art of Conquest – Strategy |
| Asphsalte 9 – Action |
| Avengers – Action |
| Back 4 Blood – Strategy & Action |
| Backgammon – Undefined |
| Ball sort puzzle – Puzzle |
| Ballsort – Puzzle |
| Battle Grounds – Unclear |
| Bejewelled – Puzzle |
| BF4 – Action |
| Bingo blitz – Undefined |
| Bish fish – Undefined |
| Bitlife – Undefined |
| Blair Witch – Strategy & Action |
| Blockscapes – Puzzle |
| Board King – Undefined |
| Bomberman – Strategy & Action |
| Brain games – Undefined |
| Brawl Stars – Action |
| Bridge – Strategy |
| Bubble pop – Puzzle |
| Bubble Shooter – Puzzle |
| Call of Duty – Strategy & Action |
| Call of Duty Black Ops Cold War – Strategy & Action |
| Call of Duty Warzone – Strategy & Action |
| Card games – Undefined |
| Castlevania: Symphony of the night - Action |
| Chef blast - Puzzle |
| Chess - Strategy |
| Chivalry 2 - Action |
| Choices - Undefined |
| Cities: Skylines – Strategy |
| City of Heroes - Action |
| Civilisation IV – Strategy |
| Civilisation V – Puzzle & Strategy |
| Civilisation VI – Strategy |
| Clash of Clans – Strategy |
| Clash Royale – Strategy |
| Clockmaker – Puzzle |
| Coin master – Puzzle |
| Colums - Puzzle |
| Cooking Diary – Strategy |
| Cooking Fever – Strategy |
| Counter Strike Global Offensive – Strategy & Action |
| Crash Bandicoot N Sane Trilogy – Strategy & Action |
| Cross Logic – Puzzle |
| Crossword – Puzzle |
| Crossy Road – Strategy & Action  Crusader Kings – Strategy |
| CS:GO – Strategy & Action |
| Daily mail – Undefined |
| Dark souls 2 : Scholar of the first sin – Strategy & Action |
| Darts – Strategy |
| Days gone – Action |
| Dead by Daylight – Undefined |
| Dead Island – Action |
| Dear My Cat – Undefined |
| Deltarune – Puzzle, Strategy & Action |
| Demos of Terra Nil – Puzzle & Strategy |
| Destiny 2 – Strategy & Action |
| Diggys Adventure – Puzzle |
| Dishonored – Strategy & Action |
| DLS – Strategy & Action |
| Dog Island – Undefined |
| Dominoes – Strategy |
| Doom Eternal – Action |
| Dota – Strategy & Action |
| Dragon Ball Fighterz – Action |
| Dragon Quest XI – Strategy & Action |
| Dragon Raja - Strategy & Action |
| Drive – Action |
| Dungeon Defenders - Strategy & Action |
| Dungeons & Dragons Online - Strategy & Action |
| Duskwood – Strategy |
| Egg Inc – Strategy |
| Elder Scrolls: Oblivion - Strategy & Action |
| Emporea – Strategy |
| Enter the gungeon – Action |
| Escaoe from Tarkov - Strategy & Action |
| Evermerge – Puzzle |
| F1 clash – Strategy |
| Faeria – Strategy |
| Fall Guys – Action |
| Fallout - Strategy & Action |
| Fallout 4 Strategy & Action |
| Fallout New Vegas - Strategy & Action |
| Farkle – Undefined  Farm King – Puzzle |
| farmville 3 – Strategy |
| Fate Grand Order – Strategy |
| Fermi Paradox – Strategy |
| Fifa - Strategy & Action |
| Final Fantasy 5 – Strategy |
| Final Fantasy XIV - Strategy & Action |
| Fit and Squeeze – Puzzle & Strategy |
| Five nights at Freddy’s - Strategy |
| Flow Bridges – Puzzle |
| Flow Fit – Puzzle |
| Flow Free – Puzzle |
| Flow Hexes – Puzzle |
| Flow Wraps – Puzzle |
| For honor – Action |
| Forager - Strategy |
| Forge of Empires – Strategy |
| Fortnite - Strategy & Action |
| Forza – Action |
| Freecell - Strategy |
| Frenzic – Undefined |
| Friday Night Funkin – Action |
| Funtrivia – Undefined |
| Futoshiki – Puzzle |
| Game of thrones casino slots – Undefined  Garry’s Mod - Undefined |
| Gauntlet – Undefined |
| Gears of War 5 – Action |
| Genshin Impact – Strategy & Action |
| Golf with Friends – Strategy |
| Grand Theft Auto 5 – Action |
| Grandia II – Puzzle & Strategy & Action |
| Gummy Drop – Puzzle |
| Hades – Strategy & Action |
| Halo CE – Action |
| Happy Color – Undefined |
| Harry Potter Puzzles & Spells – Puzzle |
| Hayday – Strategy |
| Hearthstone – Puzzle & Strategy |
| Hearts of Iron – Strategy |
| Hidden Object games – Undefined |
| Hollow Knight – Strategy & Action |
| Homescapes – Puzzle |
| House Flipper – Strategy |
| Idle games – Undefined |
| Idle Heros – Strategy |
| It takes two – Puzzle & Strategy & Action |
| Jewels of Egypt – Puzzle |
| Jewels of Rome – Puzzle |
| Jewels of the Wild West – Puzzle |
| Jigsaw puzzle – Puzzle |
| Jigsaw trainstation – Undefined |
| Jumbline – Puzzle |
| Just cause 4 – Action |
| Kards – Strategy |
| Kentucky Route Zero – Puzzle |
| Kerbal Space Program – Strategy |
| Kitchen game – Undefined |
| Klondike the lost expedition – Strategy |
| Knockout City – Action |
| Kotor – Strategy & Action |
| League of legends - Strategy & Action |
| Left 4 dead - Strategy & Action |
| Legend of Zelda: Breath of the Wild – Puzzle & Strategy & Action |
| Lego Harry Potter – Puzzle & Action |
| Lily's Garden – Puzzle |
| Little Alchemy – Undefined |
| Lost Ark – Puzzle & Strategy & Action |
| Love Nikky – Strategy |
| Ludo – Strategy |
| Madden 2 – Action  Magic Tiles 3 – Puzzle & Strategy |
| Mahjong – Undefined |
| Mario - Action |
| Mario Kart - Action |
| Mario Kart 8 Deluxe - Action |
| Mass Effect ½ - Action |
| Match 3D – Puzzle |
| Match masters – Puzzle |
| Match3 - Puzzle |
| Memory game – Undefined |
| Metroid Prime - Action |
| Miles Morales – Strategy & Action |
| Mindpal – Undefined |
| Minecraft – Strategy & Action |
| Minesweeper – Puzzle |
| Monster Hunter: World – Strategy & Action |
| Monument Valley – Puzzle |
| MTG Arena – Strategy |
| N7A – Undefined |
| Nancy Drew – Undefined |
| NBA 2k22 – Action |
| Neko Atsume – Undefined |
| Ni No Kuni II – Puzzle & Strategy & Action |
| Noita – Strategy |
| Nongram colour – Puzzle |
| Nonogram – Puzzle & Strategy |
| NSS – Strategy & Action |
| Numberzilla – Puzzle |
| Overwatch – Strategy & Action |
| Pacman – Strategy |
| Paladins – Strategy & Action |
| Papa pear saga – Puzzle |
| Path of Exile – Strategy & Action |
| Persona 5 Strikers – Action |
| Pet master – Puzzle |
| Phantome Doctrine – Strategy |
| Phasmophobia – Puzzle & Strategy & Action |
| Piano Tiles – Action |
| Picture Cross – Puzzle |
| Planet Zoo – Puzzle & Strategy |
| Pokemon Legends Arceus – Strategy & Action |
| Pokemon Mystery Dungeon – Strategy |
| Pokemon Platinum – Strategy |
| Pokemon Rescue Team – Strategy |
| PokeMon Snap – Undefined |
| Pokemon Sword – Strategy |
| Poker – Strategy |
| PONG PONG – Undefined |
| Pool – Undefined |
| Portal 2 – Puzzle & Strategy & Action |
| Professor Layton – Puzzle |
| Psychonauts – Action |
| PUBG – Action |
| Puzzle – Undefined |
| Puzzle page – Undefined |
| Puzzledom – Puzzle |
| Pyramid Solitaire - Strategy |
| Raid – Strategy & Action |
| Ratchet and Clank: Rift Apart - Action |
| Red Dead Redemption 2 – Strategy & Action |
| Ride 2 – Action |
| Rimworld – Strategy |
| Roads to Rome – Strategy |
| Roblox – Undefined |
| Robotek – Strategy & Action |
| Rocket league – Strategy & Action |
| Runescape – Undefined |
| Rust – Action |
| Satisfactory – Strategy |
| Scrabble – Puzzle |
| Sea of Thieves – Strategy & Action |
| Shape-arranging games – Undefined |
| Seaport - Undefined |
| Shin Megami Tensei III Nocturne Remastered – Strategy & Action |
| Sims 4 – Strategy |
| Simulation games – Undefined |
| Sky Children of Light – Undefined |
| Skyrim – Strategy & Action |
| Slay the Spire - Strategy |
| Slotomania - Undefined |
| Snooker 19 – Strategy |
| Soccer – Undefined |
| Solitare – Strategy |
| Sonic - Action |
| Sparkle Bubble Shooter – Puzzle |
| Spider – Undefined  Squad - Action |
| Star Realms – Strategy |
| Stardew Valley – Strategy |
| State of Decay 2 – Strategy & Action |
| Stick Cricket – Undefined |
| Stranded Deep – Strategy |
| Streets of Rage – Action |
| Stronghold – Undefined |
| Subway Surfers – Action |
| Sudoku – Puzzle |
| Sundial – Puzzle |
| Super Castlevania IV – Action |
| Super Jigsaw Puzzle – Puzzle |
| Super Mario – Strategy & Action |
| Super Mario Galaxy – Action |
| Super Smash Bros – Action |
| Surviving Mars – Strategy |
| Tabletop Simulator – Strategy |
| Tapped Out – Strategy |
| Team Fortress 2 – Strategy & Action |
| Team Sonic Racing – Action |
| Teardown – Puzzle & Action |
| Terraria – Strategy & Action |
| Tetris Puzzle & Strategy |
| The Forest – Strategy & Action |
| The Infected – Action |
| The Room – Undefined |
| The Simpsons Tapped Out – Strategy |
| The Witcher 3 – Strategy & Action |
| Thronebreaker – Strategy |
| Ticket to Ride – Strategy |
| Tile Fun – Puzzle |
| Time Princess – Strategy |
| Titanfall 2 – Strategy & Action |
| Tomb raider – Undefined |
| Township – Strategy |
| Toy Blast – Puzzle |
| Translation 2 – Undefined |
| Transport Fever 2 – Strategy |
| UFC4 – Action |
| Valheim – Strategy & Action |
| Valorant – Strategy & Action |
| War thunder – Strategy & Action |
| Warframe – Strategy & Action |
| Warpath – Strategy |
| Water Puzzle – Undefined |
| Winneroo – Undefined |
| Word Chums – Puzzle |
| Word Connect – Puzzle |
| Word connect – Puzzle |
| Word Cookies – Puzzle |
| Word Crush – Puzzle |
| Word games – Undefined |
| Word Life – Puzzle |
| Word Stacks – Puzzle |
| Word Trip – Puzzle |
| Word Wow – Puzzle |
| Wordle – Puzzle |
| Wordlink – Puzzle |
| Words with friends – Puzzle |
| Wordscapes – Puzzle |
| World Golf Tour – Strategy |
| World of Tanks - Strategy & Action |
| Wreckfest – Action |
| Yakuza – Strategy & Action |
| Yakuza 5 - Action |
| Yu Gi Oh Link Evolution – Strategy |
| Yu Gu Oh! Master Duel – Strategy |
| Zoo Keeper – Undefined |
